# Understanding Colloidal Quantum Dot Device Characteristics with a Physical Model

**DOI:** 10.1021/acs.nanolett.3c02899

**Published:** 2023-10-24

**Authors:** Shaurya Arya, Yunrui Jiang, Byung Ku Jung, Yalun Tang, Tse Nga Ng, Soong Ju Oh, Kenji Nomura, Yu-Hwa Lo

**Affiliations:** † Department of Electrical and Computer Engineering, University of California San Diego, La Jolla, California 92093, United States; ‡ Department of Materials Science and Engineering, Korea University, Seoul 02841, Republic of Korea

**Keywords:** colloidal quantum dots (CQDs), lead sulfide (PbS), heterojunction, solar cell, photodetection, physical model

## Abstract

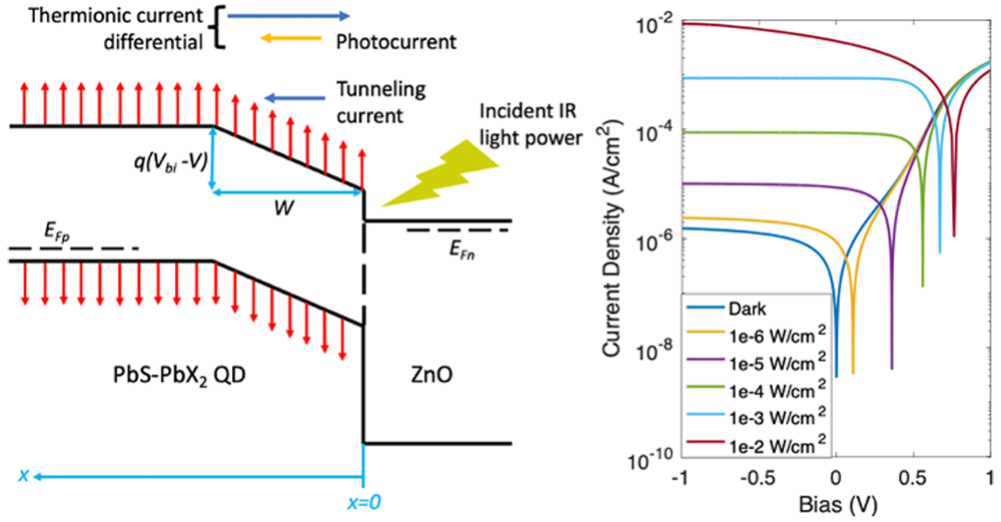

Colloidal quantum dots (CQDs) are finding increasing applications in optoelectronic devices, such as photodetectors and solar cells, because of their high material quality, unique and attractive properties, and process flexibility without the constraints of lattice match and thermal budget. However, there is no adequate device model for colloidal quantum dot heterojunctions, and the popular Shockley–Quiesser diode model does not capture the underlying physics of CQD junctions. Here, we develop a compact, easy-to-use model for CQD devices rooted in physics. We show how quantum dot properties, QD ligand binding, and the heterointerface between quantum dots and the electron transport layer (ETL) affect device behaviors. We also show that the model can be simplified to a Shockley-like equation with analytical approximate expressions for reverse saturation current, ideality factor, and quantum efficiency. Our model agrees well with the experiment and can be used to describe and optimize CQD device performance.

Colloidal quantum dots (CQDs) have attracted significant attention in recent years because of their unique properties and wide-ranging applications in electronics,^1^ optoelectronics,^2^ biology,^3^ and energy.^4^ In contrast with conventional epitaxial growth, CQDs can be deposited on many substrates at low temperature without the constraints of a lattice match and thermal budget. These nanoscale semiconductor particles exhibit quantum confinement effects, which leads to discrete energy levels and size-dependent optical and electronic properties.^5,6^ Because of the low-temperature solution-based synthesis process governed by thermodynamics, CQDs can obtain high material quality with a low density of dislocations and point defects. Another key advantage of colloidal quantum dots is their tunable band gap, which enables precise control over their absorption and emission wavelengths.^7−11^ The above features make CQDs highly desirable for applications, such as light-emitting diodes (LEDs),^12^ photodetectors,^13,14^ solar cells,^15^ and biomedical imaging.^16^

Many groups have demonstrated CQD photodetectors with high sensitivity,^17,18^ fast response times,^18^ and wavelength selectivity. For example, lead sulfide (PbS) quantum dots, with their strong light absorption coefficient (in the order of 10^5^–10^6^ cm^–1^)^19^ in the infrared region, have shown excellent quantum efficiency for infrared photodetection, thereby showing great promise for applications in night vision,^20^ environmental sensing,^21^ and communications.^22^ CQDs are also finding significant application in solar cells by overcoming the limitations of traditional photovoltaic technologies.^5,9^ The size-dependent band gap of CQDs allows for the utilization of a broader spectrum of sunlight to enhance the overall efficiency of the solar cell. Detectors and solar cells are just two of many examples where CQDs offer unique advantages, and new applications of CQDs continue to emerge.

While the experimental advancements in CQD-based devices have been remarkable, the theoretical understanding of their behaviors has not kept up with technology development. As a result, the characteristics of CQD devices can be modeled only by empirical formulas with fitting parameters. However, theoretical models rooted in physics are essential to realize the full potential of CQDs. A device physics model helps to interpret experimental results and provides a deep understanding of the underlying mechanisms at play. It helps identify the dominant processes that limit device performance and provides guidelines for mitigating such limitations, which allows researchers to predict and develop strategies to optimize device performance.

While there has been some work done in this regard,^23−25^ researchers by and large use the Shockley–Queisser diode model^26^ to describe the behavior of the QD-based devices. However, unlike conventional semiconductor junctions from which the Shockley–Queisser (S-Q) diode model was derived, carrier transport mechanisms for ligand-bonded quantum dots are fundamentally different from the S-Q diode model.^27,28^ Although it has become a common practice for researchers to use the S-Q diode model to describe the *I*–*V* relation and extract parameters, such as quantum efficiency, ideality factor, reverse saturation current, open-circuit voltage, short-circuit current, etc., here, the S-Q model is treated as an empirical model for curve-fitting purposes without offering any insight into device characteristics.

In this work, we derive the first physics model for a CQD heterojunction and connect those popular parameters, such as ideality factor, reverse saturation current, etc., to the material properties. Our model can be generally applied to different types of CQDs and heterojunctions and finds good agreement with experimental results. We show that the **I–V** characteristics of a CQD heterojunction depend on four parameters: effective inter-QD tunneling barrier, built-in potential, carrier lifetime, and electron density at the heterointerface. We also show that an approximate expression similar to the Shockley diode equation can be derived from our model after some approximations, although the key parameters in the expression, such as the reverse saturation current and ideality factor, possess completely different physical origins from the S-Q model.

For illustration purposes, we modeled a PbS-EDT/PbS-PbX_2_/ZnO heterostructure, paying special attention to the PbS-PbX_2_/ZnO heterointerface. The PbS QDs connected with ethane-1,2-dithiol (EDT) ligands form the p-type electron-blocking layer (EBL),^29^ and the PbS CQDs with halide ligands represented by PbX_2_ are the light-absorber layer and form a heterointerface with ZnO (n-type semiconductor). All the results from this analysis, however, are general and can be readily applied to other CQD heterojunctions, as well.

The key idea of the model is to use the two physical processes of thermionic emission and tunneling^30,31^ to model the transport of carriers across the CQD layer. Focusing on the electron transport in CQDs, the electron current density due to thermionic emission from one quantum dot to another is given by
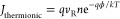
1where *q* is the elementary charge,  is the Richardson velocity, *n* is the electron concentration, and ϕ is the barrier height (in Volts), which is determined by the ligands bound to the quantum dots. The detailed derivation of eq 1 and other equations in this section can be found in the Supporting Information.

Since thermionic emission does not depend on the electric field, we need to consider both positive and negative directions for the flow of electrons, which leads to a net thermionic differential current
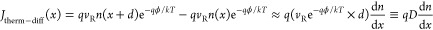
2where *d* is the center-to-center distance between two quantum dots, which is equal to the quantum dot diameter plus the ligand length. The form of eq 2 is analogous to a diffusion current given by Fick’s law. Therefore, we can define an effective diffusion constant, , which is a function of the barrier height.

Next, we use the Fowler–Nordheim model for quantum mechanical tunneling
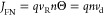
3where  is the transmission probability, and *E* is the electric field. In the following, we write Θ = e^–*B*/*E*^ for simplicity. In eq 3, we define the effective drift velocity *v*_d_ = *v*_R_e^–*B*/*E*^. It should be noted that we are not using the concept of mobility here because of the nonlinear dependence of drift velocity on the electric field.

Adding eqs 2 and 3 gives us the total electron current density.
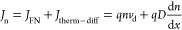
4

We first analyze the equilibrium scenario using eq 4. Assuming zero current and using the equilibrium electron concentration for a nondegenerate semiconductor (*n* = *n*_i_e^(*E*_F_ – *E*_i_)/*kT*^), we can conclude that the electric field at any position in the CQD layer can be either zero or a constant value given by
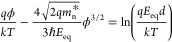
5where *E*_eq_ is the nonzero equilibrium electric field and is a function of the barrier height. Figure 1a depicts the band bending at equilibrium. The width of the band bending region is given by *W* = *V*_bi_/*E*_eq_ where *V*_bi_ is the built-in potential in the CQD layer. *V*_bi_ depends on the bulk carrier concentrations in PbS-PbX_2_ and zinc oxide (ZnO) and the difference between their conduction band minima (Δ*E*_C_). In other words, *qV*_bi_ = Φ_CQD_ – Φ_ZnO_ – Δ*E*_C_, where Φ represents the work functions of the CQD layer and ZnO. In the following analysis, we focus on the band bending region of width *W* that contributes to the carrier transport. Here, we have assumed that ZnO has high enough doping or interface states such that the band bending in ZnO is negligible. Finally, we can write the equilibrium electron concentration as a function of position *x*
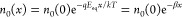
6where we define β = *qE*_eq_/*kT*, and *n*_0_(0) is the electron concentration in PbS-PbX_2_ at the heterointerface with the ZnO electron transport layer (ETL) layer. This quantity *n*_0_(0) depends on the ZnO doping and the interface charge. We simply use *n*_0_ to refer to *n*_0_(0) in the following.

**Figure 1:**
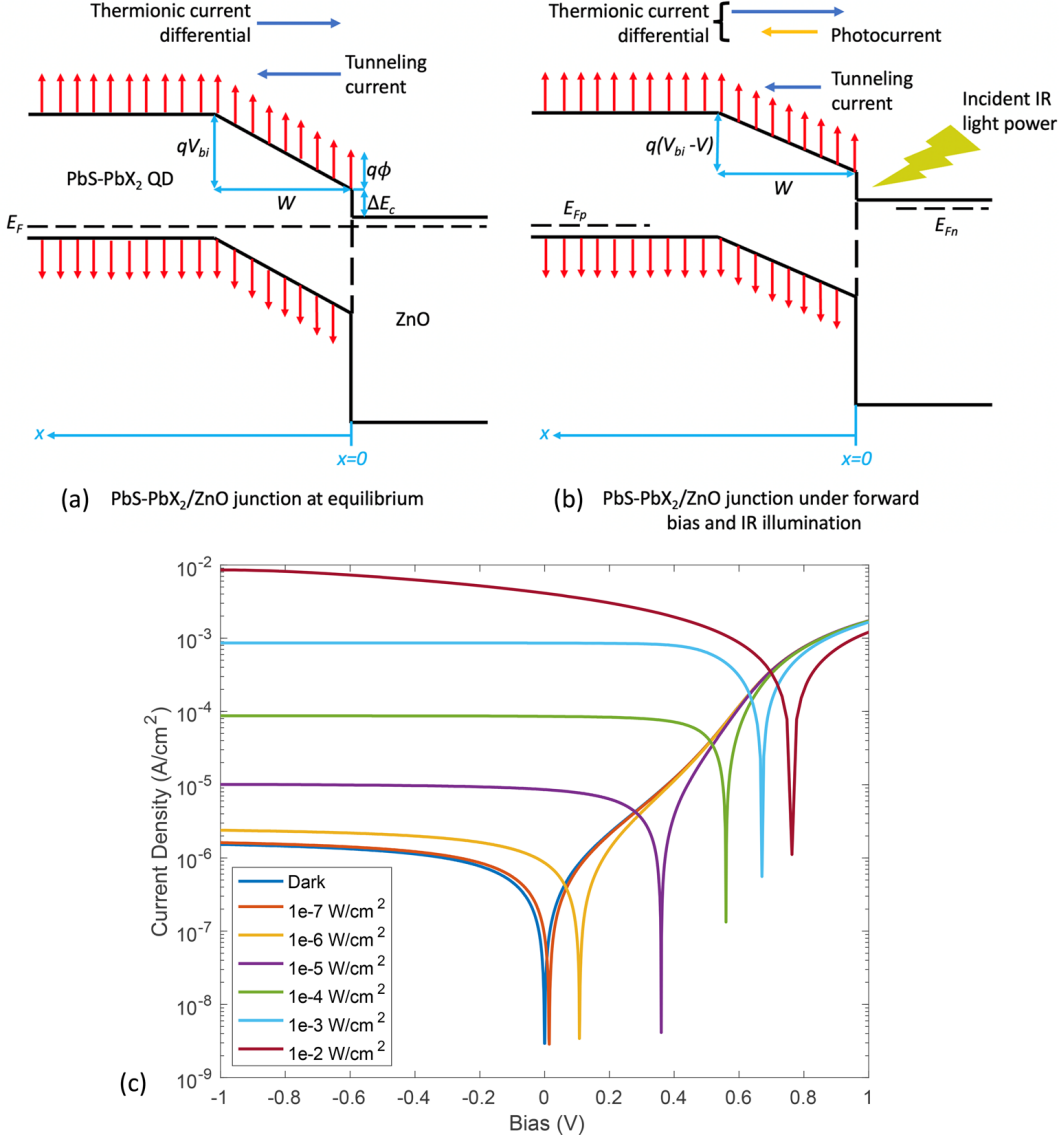
Schematic band diagram and *I–V* characteristics. (a) Band diagram of the CQD heterojunction at equilibrium. Note that the band bending region has a constant *E* field, as given by eq 5. (b) Band diagram of the CQD heterojunction under applied forward bias and light illumination. (c) An example of the dark and illuminated *I–V* curves calculated from the theoretical model.

Next, we analyze the device behavior under an applied bias and incident light. The steady-state continuity equation is given by
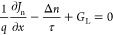
7where τ is the electron carrier lifetimen and *G*_L_ is the carrier generation rate due to incident light.

For normal light incidence from the ZnO end of the device, *G*_L_ can be written as
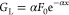
8where α is the absorption coefficient of the CQD layer at the wavelength of incident light, and *F*_0_ is the photon flux (number of photons per second per cm^2^).

Substituting eqs 4, 6, and 8 in eq 7, we obtain
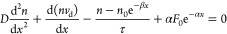
9In order to solve eq 9 analytically, we use the following assumption. The width of the band bending region does not change with bias significantly, and the electric field in the band bending region is given approximately by *E* = (*V*_bi_ – *V*)/*W*, where *V* is the applied bias. This is depicted in Figure 1b. The detailed explanation for this assumption is given in the Supporting Information.

With this approximation, drift velocity (*v*_d_) becomes position-independent, and eq 9 can be rewritten as
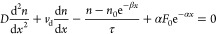
10

The general solution of eq 10 is given by
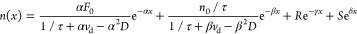
11where , , and *R* and *S* are constants dependent on boundary conditions. We conclude that *S* = 0 since *n*(*x* = ∞) cannot be infinitely large. Here, we make another assumption: the electron density at the interface of QD and ZnO, *n*(0), is nearly unchanged with current.

This can be justified since its value is determined primarily by the property of the ZnO layer and the ZnO/CQD interface. Through this assumption, *R* in eq 11 can be obtained using the boundary condition *n*(*x* = 0) = *n*_0_. A detailed explanation for this assumption is given in the Supporting Information.

Substituting the result from eq 11 into eq 4 and using the relation *J* = −*J*_n_(*x* = 0) for the total current density at the PbS and ZnO interface,
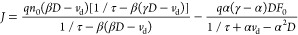
12It should be noted that the negative sign in front of *J*_n_(*x* = 0) is because of our choice of *x* coordinate direction, which is opposite to the conventional choice (p-side to n-side). Equation 12 represents the direct current behavior of the device under illumination. The first term is the dark current, and the second term is the photocurrent. We do not need to do a separate analysis for the transport of holes since the hole current flowing from PbS-PbX_2_ to ZnO is negligible at the heterointerface [i.e., *J*_p_(*x* = 0) = 0]. This is because of the high barrier created by the valence band maximum (VBM) of the ETL (ZnO in this case) and makes sure that almost no holes cross the interface. Although the hole current is zero near the interface, it is nonzero at other *x* > 0 and can be evaluated using *J*_p_(*x*) = *J* – *J*_n_(*x*).

Figure 1c shows an example of the *I–V* characteristics obtained under dark and different light irradiance conditions. The list of parameters used in Figure 1c and other subsequent figures is provided in the Supporting Information.

The first term in eq 12 gives the dark current density.
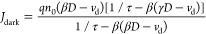
13where *n*_0_ is the electron density at the PbS QD/ZnO heterointerface, and τ is the electron carrier lifetime determined by both the material quality of QDs and the PbS QD/ZnO heterointerface. β = *qE*_eq_/*kT* is a quantity determined by the barrier height (ϕ) through eq 5. Similarly, an effective diffusion constant,  depends on ϕ. The bias dependence is captured in the effective drift velocity, *v*_d_ = *v*_R_ exp[−*BW*/(*V*_bi_ – *V*)], and consequently, , which also depends on built-in voltage (*V*_bi_), ϕ, and τ. It should be noted that 1/γ represents the effective diffusion length. Finally, to include the effect of any series resistance (due to ZnO and other factors), we can substitute *V* in eq 13 with *V* – *J*_dark_*R*_S_, where *R*_S_ is the area-normalized series resistance (in Ω cm^2^). The series resistance, however, only affects the dark current at high biases and has no effect on open-circuit voltage (*V*_oc_). To summarize, the device *I–V* characteristics depend on four key parameters: tunneling barrier height (ϕ), built-in potential (*V*_bi_), carrier lifetime (τ), and the electron density at the heterointerface (*n*_0_). Next, we analyze how each of the four parameters affects the device performance by investigating the dependence of dark current and open-circuit voltage (*V*_oc_) on them.

The tunneling barrier height (ϕ) depends on the ligand passivation of quantum dots. The ligand structure and bonding are the primary factors that affect ϕ. Recent studies have also shown that ligands might undergo changes in bonding during the excitonic excited states of the QD,^32^ thereby leading to a smaller tunneling barrier. Figure 2a shows the dark current dependence on the barrier height under forward and reverse bias. Note that there is not a simple relation between current and barrier height under a given bias voltage. As discussed previously, the net current is determined by the difference between thermionic emission current and tunneling current. While the thermionic emission current changes exponentially with the barrier height, the tunneling current has a more complicated dependence on the barrier height governed by the Fowler–Nordheim model. This leads to a complicated relationship between barrier height and *I–V* characteristics. Among the key features in the *I–V* characteristics, the forward current saturates with bias voltage, and the magnitude of forward saturation current increases with reduced barrier height. However, under low forward bias voltage, the forward current increases more rapidly as the barrier height increases and reaches its saturation level at lower bias voltage. The reverse bias current, in contrast, shows weak dependence on barrier height. However, when the barrier height is reduced to a very low value, the reverse saturation current is lowered significantly in favor of detector operation for low dark current. Figure 2b shows the photoresponse in terms of the open-circuit voltage dependence (*V*_oc_) on the irradiance. Under a given irradiance, the *V*_oc_ decreases when the barrier height increases. The results in Figures 2a,b agree with studies that have shown that increasing ligand passivation by employing two-step instead of one-step ligand-exchange^33^ or by using a different ligand (methylammonium acetate vs ammonium acetate)^34^ leads to an increase in forward bias current.

**Figure 2:**
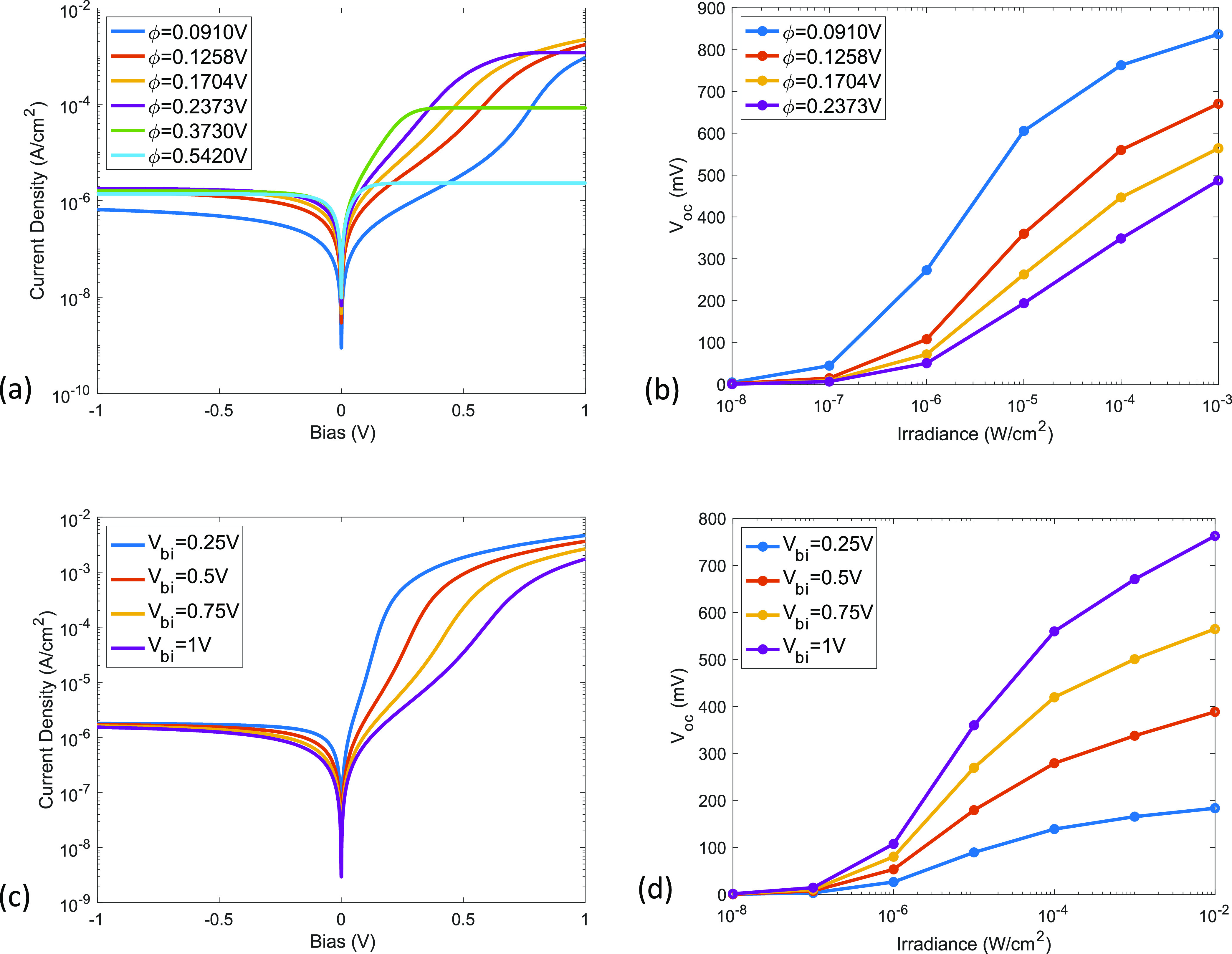
Effect of barrier height and built-in potential on dark current and open-circuit voltage. (a) Dark current density variation with barrier height. (b) Open-circuit voltage variation with barrier height. (c) Dark current density variation with built-in potential. (d) Open-circuit voltage variation with built-in potential. The results show that the best device performance (i.e., low reverse saturation current and high *V*_oc_) is obtained with reduced barrier height and high built-in potential.

Built-in potential (*V*_bi_) is another key parameter for CQD heterojunction devices. The built-in potential depends on the work function difference and band offset between PbS-PbX_2_ and zinc oxide (ZnO). Figure 2c shows that decreasing *V*_bi_ leads to a faster increase in current at small forward biases, while the reverse bias current shows negligible dependence on *V*_bi_. The open-circuit voltage shows a linear dependence on *V*_bi_ in Figure 2d. A study on the PbS-QD/n-Si heterojunction photodetector has shown that increasing the doping level of silicon (from light- to medium-doped) lowered the forward bias current but kept the reverse bias current almost the same.^35^ This is consistent with our model since a doping level increase in the layer next to the QDs will increase the built-in potential.

The minority (electron) carrier lifetime (τ) in PbS QDs primarily depends on the radiative and nonradiative exciton recombination dynamics and their ionization rate under an electric field. Factors that affect τ include delocalization in intermediate bands,^36^ ligand properties,^37,38^ and trap states.^39^ As shown in Figure 3a, the reverse saturation current is nearly inversely proportional to the electron lifetime, which is analogous to the reverse current because of recombination–generation in a nonideal diode vis-à-vis the 1/√τ dependence for an ideal diode. This also leads to the rightward shift of the *V*_oc_ vs irradiance curve in Figure 3b.

**Figure 3:**
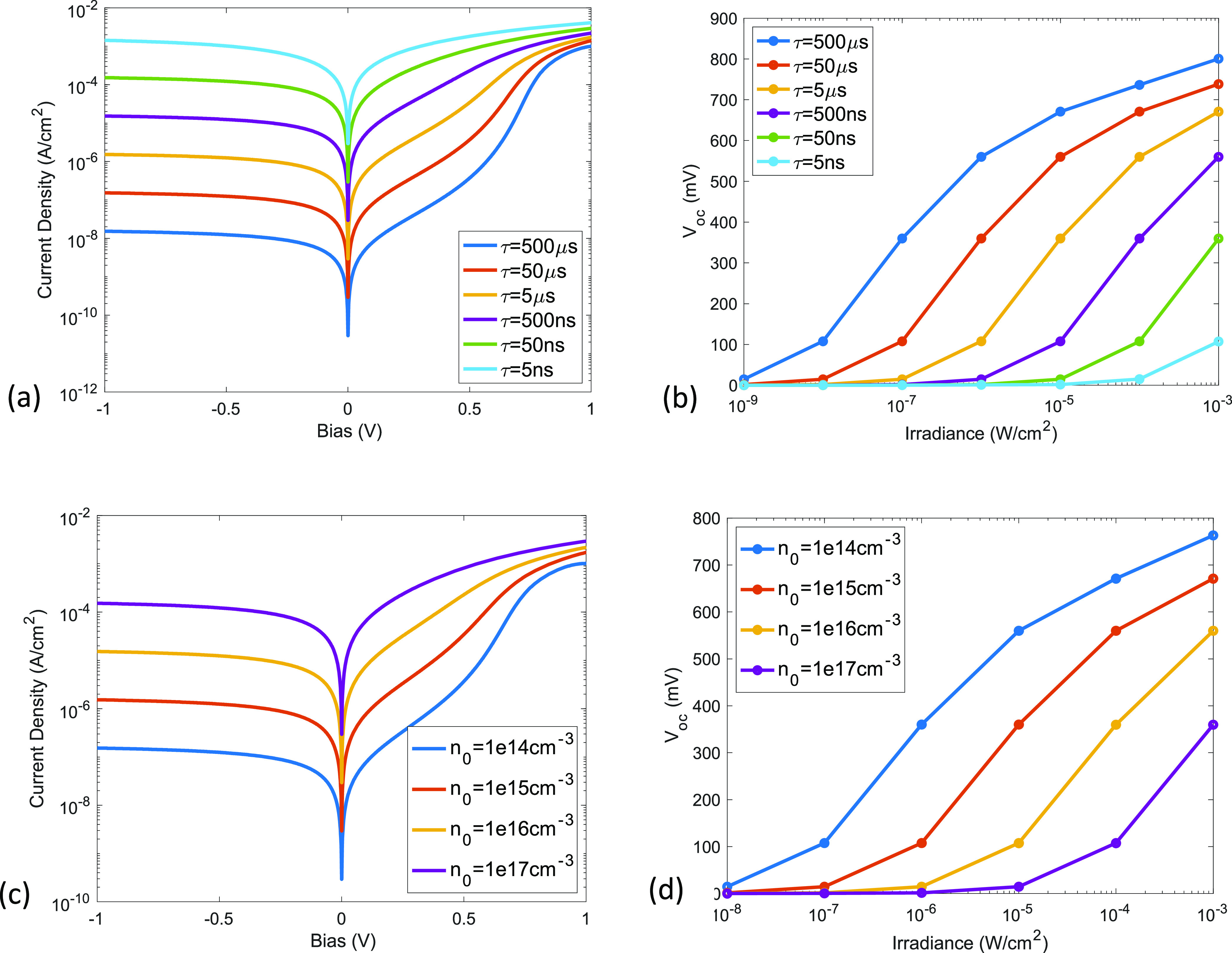
Effect of carrier lifetime and interface electron density on dark current and open-circuit voltage. (a) Dark current density variation with carrier lifetime. (b) Open-circuit voltage variation with carrier lifetime. (c) Dark current density variation with interface electron density. (d) Open-circuit voltage variation with interface electron density.

The electron density in PbS-PbX_2_ at the heterojunction (*n*_0_) is expected to depend on the ZnO doping level and the interface charge at the PbS/ZnO heterointerface. The dark current from eq 13 is directly proportional to *n*_0_, and therefore, we get the results shown in Figure 3c,d. The aforementioned study about PbS-QD/n-Si photodetector also shows that the dark current increases with Si doping level (medium to highly doped).^35^ This result can be explained by our model since the increase in *V*_bi_ with a higher doping level is no longer the dominant effect, and the increase in *n*_0_ leads to an increase in dark current in both forward and reverse bias.

Although eq 12 captures the full device behavior, it is nonetheless beneficial to have a simpler expression. Using some approximations, we can reduce eq 12 to a Shockley-like diode equation
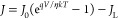
14where *J*_0_ is the reverse saturation current density, η is the effective ideality factor, and *J*_L_ is the photocurrent. *J*_L_ can be expressed as *J*_L_ = *qF*_0_(*QE*) where *QE* is the internal quantum efficiency of the device. Next, we analyze how the reverse saturation current density, effective ideality factor, and quantum efficiency in eq 14 depend on the four key parameters (ϕ, *V*_bi_, τ, and *n*_0_) discussed previously.

After making some approximations, we can express the reverse saturation current density as
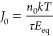
15Equation 15 shows that *J*_0_ primarily depends on τ and *n*_0_ and very weakly on ϕ, since *E*_eq_ varies slowly with ϕ from eq 5. The simplified model agrees well with Figure 3a,d, which shows that *J*_0_ is directly proportional to *n*_0_ and nearly inversely proportional to τ. Similarly, the weak dependence on ϕ agrees with the result from Figure 2a.

The effective ideality factor can be represented as
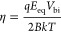
16where . The ideality factor is directly proportional to *V*_bi_ and decreases with increasing ϕ, which is also observed in Figure 2a–d. As shown in Figure 4b, η can take values greater than 2, which was reported in previous studies.^40,41^ The value of the ideality factor is determined by the underlying physics of carrier transport in QDs. We use the term “ideality factor” here only for namesake, and it does not imply any ideal vs nonideal behavior of the PbS-QD/ZnO heterojunction.

**Figure 4:**
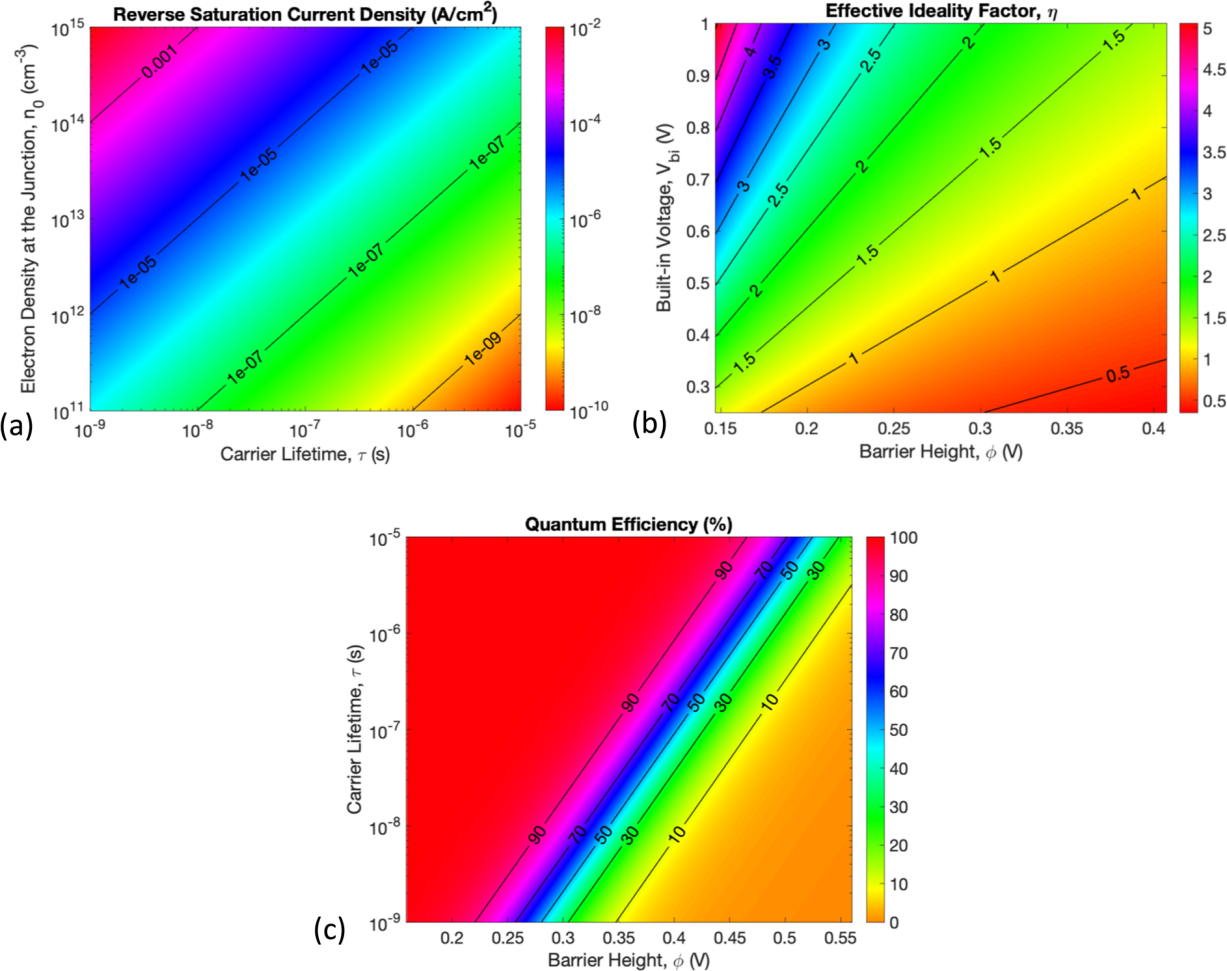
Variation of device performance parameters. (a) Effect of interface electron density and carrier lifetime on reverse saturation current density. (b) Effect of built-in potential and barrier height on the effective ideality factor. (c) Effect of carrier lifetime and barrier height on internal quantum efficiency.

The quantum efficiency can be represented as
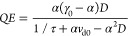
17where *v*_d0_ = *v*_R_ e^–*B*/*E*_eq_^ is the zero-bias (equilibrium) drift velocity and . Hence, *QE* depends on ϕ and τ, although it is not apparent from eq 17. Figure 4c shows that increasing carrier lifetime and decreasing barrier height significantly increases the quantum efficiency. These results are backed by other works, as well.^33,34^ For easy reference, the key expressions from the model are summarized in Table 1.

**Table 1:** Summary of Key Expressions and Results from the Model

input parameters for the model: built-in potential (***V***_**bi**_), tunneling barrier height (**ϕ**), carrier lifetime (**τ**), electron density at the heterointerface (***n***_**0**_)	
these parameters are in bold letters when they appear in the expressions below	
built-in Voltage (*V*_bi_)	*q**V***_**bi**_ = Φ_CQD_ – Φ_ZnO_ – Δ*E*_C_
equilibrium electric field (*E*_*eq*_)	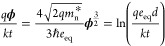
effective drift velocity (*v*_*d*_)	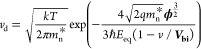
effective diffusion coefficient (*D*)	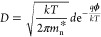
width of band-bending region (*W*)	*W* = ***V*_bi_**/*E*_eq_
effective diffusion length (1/γ)	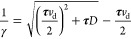
β	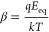
current density (*J*)	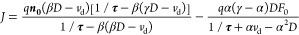
	note: to include area-normalized series resistance (*R*_*s*_), replace *V* with *V* – *JR*_s_ in the expression for *v*_d_

simplified model	
current density (*J*)	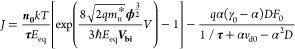
reverse saturation current density (*J*_0_)	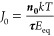
effective ideality factor (η)	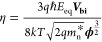
internal quantum efficiency (*QE*)	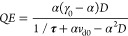

To experimentally verify the model, we fabricated devices with the structure PbS-EDT/PbS-PbX_2_/ZnO, which had the absorption peak of PbS QDs at 1064 nm. The details of quantum dot synthesis, device fabrication, and additional experimental data can be found in the Supporting Information. Figure 5a shows that the dark current derived from the theoretical model in eq 13, and the simplified expression in eqs 14–16 agree well with the experimental result in the forward bias. From Figure 5a and additional experimental data in Supporting Information, we find that the root-mean-square deviation between the calculated and measured forward bias current is in the range 1.41–74.7 μA/cm^2^. The model appears to underestimate the voltage dependence of the reverse bias current. The discrepancy between the theory and experiment in the reverse bias regime can be attributed to leakage current and extra mechanisms, such as carrier multiplication reported in the literature^42−44^ but not included in our model. Figure 5b shows the comparison of photoresponse (open-circuit voltage, *V*_oc_) between experiment and calculations by the model (eq 13) and the simplified expression (eqs 14–16). The theoretical model agrees well with the experiment, with a root-mean-square deviation between the calculated and measured *V*_oc_ values in the range of 7.4–25.6 mV, but the simplified expression that fits the mathematical form of the Shockley diode model shows appreciable deviations from the experiment. The result suggests that one should be careful to use the Shockley diode model to fit the photoresponse of a CQD heterojunction. However, the simplified expression can be used to fit the forward bias characteristics from which one can extract the analytical relations between the material properties (ϕ, *n*_*o*_, *V*_bi_, τ) and *J*_o_ and η from Figure 4 or eqs 15 and 16. Additionally, Figure 5c shows *I–V* curves under different irradiances. Figure 5c shows that the model agrees well with the experimental data for the open-circuit voltage and short-circuit current under various irradiances, except for the irradiance of 1.88 × 10^–3^ W/cm^2^, which somehow shows a greater deviation than the rest.

**Figure 5:**
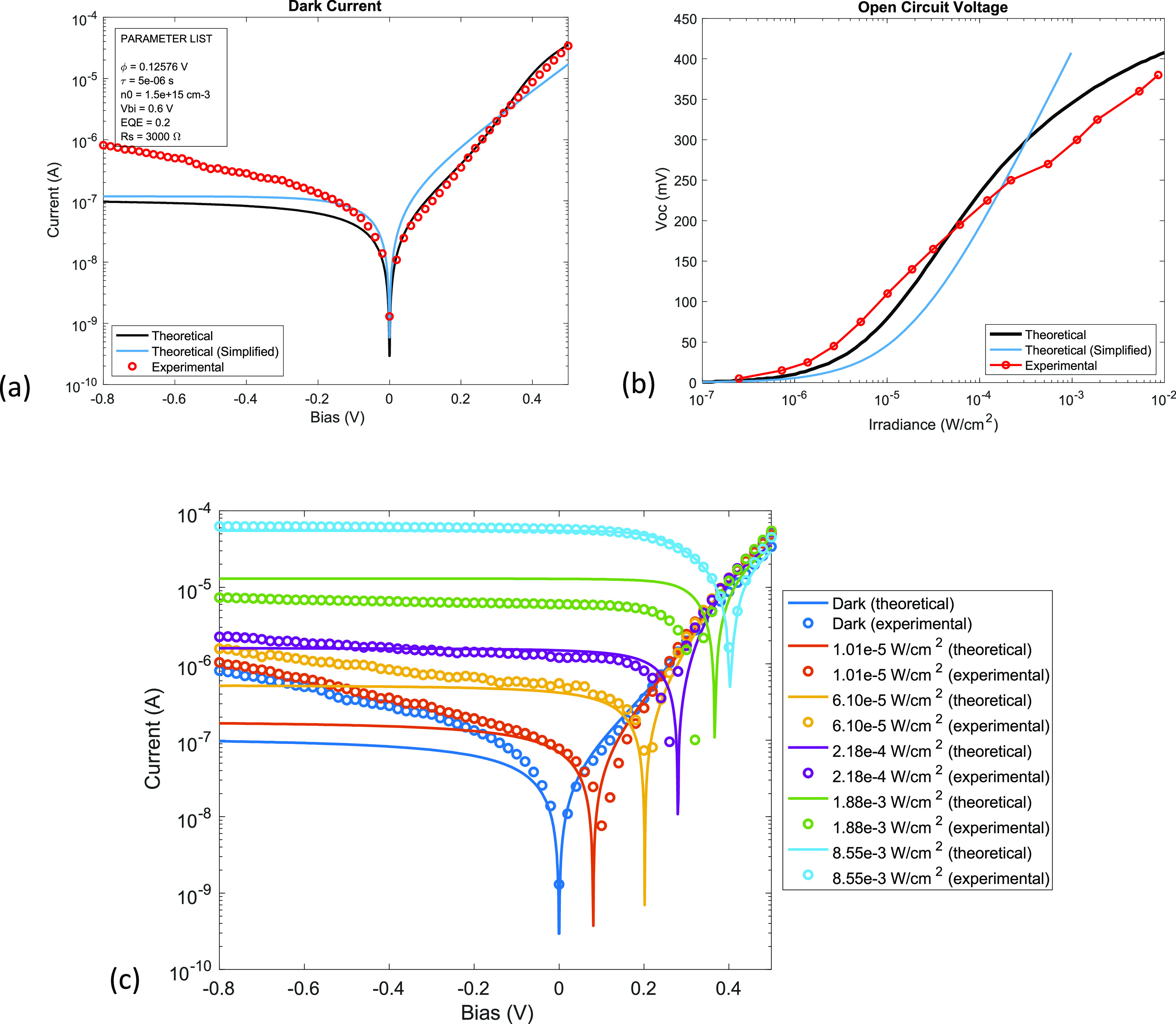
Experimental verification of the model. (a) Experimental dark current curve with the theoretical model and its simplified expression. (b) Experimental and theoretical open-circuit voltages under varying irradiance. The simplified (Shockley-like) model shows appreciable deviations from the experiment. (c) Experimental and theoretical *I–V* curves under different irradiances.

In conclusion, we have developed the first model for CQD heterojunction devices on the basis of device physics, thereby filling a major gap in the development of colloidal quantum dot devices that have found broad applications in optoelectronics. Using one of the most common CQD heterojunctions, PbS-EDT/PbS-PbX_2_/ZnO, as an example, we show that the *I–V* characteristics can be succinctly understood from four parameters: tunneling barrier height, built-in potential, carrier lifetime, and electron density at the heterointerface. We show that the underlying transport mechanisms between quantum dots are thermionic emission and tunneling, which is fundamentally different from carrier diffusion and drift in a conventional p/n junction. Therefore, the popular Shockley diode model does not apply to CQD heterojunctions. However, we show that after some approximations, we can simplify our model to obtain an expression similar to the Shockley diode equation, albeit with different underlying physics behind those parameters (reverse saturation current, ideality factor, and quantum efficiency). We have also derived analytic expressions for the reverse saturation current, ideality factor, and quantum efficiency. The model is general and can be applied to different CQD heterojunctions to describe device behaviors and relate device performance to material properties and device structures.

## Supporting Information Available

Detailed derivations, list of parameters, additional experimental details, materials, and methods (PDF)
