# Supplementary material for: Understanding Colloidal Quantum Dot Device Characteristics with a Physical Model
Source: Nano Lett. 2023 Oct 24;23(21):9943–52. doi: 10.1021/acs.nanolett.3c02899 (PMC10636828; doi:10.1021/acs.nanolett.3c02899)
Supplement: Supplementary file 1 — nl3c02899_si_001.pdf [file nl3c02899_si_001.pdf]

Understanding Colloidal Quantum Dot Device Characteristics with a Physical Model: Supplemental Document

Shaurya Arya[1], Yunrui Jiang[1], Byung Ku Jung[2], Yalun Tang[1], Tse Nga Ng[1], Soong Ju Oh[2], Kenji Nomura[1], and Yu-Hwa Lo[1]*

[1]Department of Electrical and Computer Engineering, University of California San Diego, CA 92093, USA

[2]Department of Materials Science and Engineering, Korea University, Seoul 02841, Republic of Korea

Derivation of Thermionic Emission Current

The thermionic emission theory posits that electrons with energies exceeding the height of a barrier can cross the barrier if they are directed towards it. In this theory, the specific shape of the barrier is disregarded. The electron current density in one direction is given by

$$J_+ = \int_{E_c+\phi}^{\infty} q v_x \frac{dn}{dE} dE \qquad (S1)$$

where E_c is the CQD conduction band minimum, ϕ is the barrier height due to binding ligands, q is the elementary charge, v_x is the velocity of electron in the x-direction, n is the electron density and E is the energy of the electron. Assuming non-degeneracy,

$$\frac{dn}{dE} = g(E)f(E) = \frac{4\pi\left(2m_n^*\right)^{\frac{3}{2}}}{h^3}\sqrt{E-E_c}\, e^{(E_F-E)/kT} \qquad (S2)$$

where $g(E)$ is the conduction band density of states, $f(E)$ is the Fermi function, m_n^* is the electron effective mass. Since electrons with high enough energy for thermionic emission are not quantum confined, we can use the 3D density of states as above. Assuming parabolic band (i.e., $E - E_c = m_n^* v^2/2$),

$$\frac{dn}{dE}dE = 2\left(\frac{m_n^*}{h}\right)^3 e^{(E_F - E_c)/kT} e^{-m_n^* v^2/2kT} 4\pi v^2 dv \qquad (S3)$$

Using $v^2 = v_x^2 + v_y^2 + v_z^2$, (S1) can be written as

$$J_+ = 2\left(\frac{m_n^*}{h}\right)^3 e^{\frac{E_F - E_c}{kT}} \int_{\sqrt{2q\phi/m_n^*}}^{\infty} q v_x e^{-\frac{m_n^* v_x^2}{2kT}} dv_x \int_{-\infty}^{\infty} e^{-\frac{m_n^* v_y^2}{2kT}} dv_y \int_{-\infty}^{\infty} e^{-\frac{m_n^* v_z^2}{2kT}} dv_z \qquad (S4)$$

Evaluating the integrals in (S4),

$$J_+ = 2q\left(\frac{m_n^*}{h}\right)^3 e^{\frac{E_F - E_c}{kT}} \times \frac{kT}{m_n^*} e^{-\frac{q\phi}{kT}} \times \frac{2\pi kT}{m_n^*} = q\sqrt{\frac{kT}{2\pi m_n^*}}\left[2\left(\frac{2\pi m_n^* kT}{h^2}\right)^{\frac{3}{2}} e^{\frac{E_F - E_c}{kT}}\right] e^{-\frac{q\phi}{kT}} \qquad (S5)$$

where $v_R = \sqrt{\frac{kT}{2\pi m_n^*}}$ is the Richardson velocity and $n = 2\left(\frac{2\pi m_n^* kT}{h^2}\right)^{3/2} e^{\frac{E_F - E_c}{kT}}$ is the expression for electron density. Hence, (S5) can be written as

$$J_{thermionic} = q v_R n e^{-\frac{q\phi}{kT}} \qquad (S6)$$

Equilibrium Electric Field Variation with Barrier Height

Total electron current density is expressed as the sum of the thermionic differential current and the tunneling current.

$$J_n = J_{FN} + J_{therm-diff} = qnv_d + qD\frac{dn}{dx} \qquad (S7)$$

where $v_d = v_R \exp\left(-\frac{4\sqrt{2qm_n^*}}{3\ \hbar}\frac{\phi^{\frac{3}{2}}}{E}\right) = v_R e^{-B/E}$ is the effective drift velocity, E is the electric field, $D = v_R d e^{-\frac{q\phi}{kT}}$ is the effective diffusion constant and d is the center-to-center distance between QDs. At equilibrium, $J_n = 0$ and electron density, $n = n_i e^{(E_F - E_i)/kT}$. Thus,

$$\frac{dn}{dx} = -\, n_i e^{\frac{E_F - E_i}{kT}} \frac{dE_i}{dx} = -qnE_{eq}(x) \qquad (S8)$$

where $E_{eq}(x)$ is the equilibrium electric field. Substituting (S8) in (S7),

$$0 = qnv_R \exp\left(-\frac{4\sqrt{2qm_n^*}}{3\ \hbar}\frac{\phi^{\frac{3}{2}}}{E_{eq}(x)}\right) - q^2 v_R d e^{-\frac{q\phi}{kT}} nE_{eq}(x) \qquad (S9)$$

From (S9), we can conclude that $E_{eq}(x)$ is either zero or a constant value given by the following expression

$$\frac{q\phi}{kT} - \frac{4\sqrt{2qm_n^*}}{3\hbar E_{eq}}\phi^{\frac{3}{2}} = \ln\left(\frac{qE_{eq}d}{kT}\right) \qquad (S10)$$

(S10) is an implicit expression explaining the dependence of E_{eq} on ϕ. Computationally, it is easier to calculate the value of ϕ for a given value of E_{eq} since (S10) represents a cubic equation in $\sqrt{\phi}$.

Solution of the Continuity Equation

The steady-state continuity equation for electrons under applied bias and light illumination is given by

$$D\frac{d^2n}{dx^2} + v_d\frac{dn}{dx} - \frac{n - n_0 e^{-\beta x}}{\tau} + \alpha F_0 e^{-\alpha x} = 0 \qquad (S11)$$

where n_0 is the electron concentration in CQD layer at the heterojunction, β is defined as $\beta = qE_{eq}/kT$, τ is the minority carrier lifetime, α is the light absorption coefficient and F_0 is the photon flux. Solution for $n(x)$ is of the form

$$n(x) = Pe^{-\alpha x} + Qe^{-\beta x} + Re^{-\gamma x} + Se^{\delta x} \qquad (S12)$$

where $\gamma = \frac{v_d}{2D} + \sqrt{\left(\frac{v_d}{2D}\right)^2 + \frac{1}{\tau D}}$ and $\delta = -\frac{v_d}{2D} + \sqrt{\left(\frac{v_d}{2D}\right)^2 + \frac{1}{\tau D}}$. $S = 0$ since $n(x)$ cannot go to infinity as x increases. Substituting (S12) in (S11) –

$$\begin{aligned} 0 &= D\left(\alpha^2 Pe^{-\alpha x} + \beta^2 Qe^{-\beta x} + \gamma^2 Re^{-\gamma x}\right) - v_d\left(\alpha Pe^{-\alpha x} + \beta Qe^{-\beta x} + \gamma Re^{-\gamma x}\right) - 1/\tau \\ &\quad \left(Pe^{-\alpha x} + Qe^{-\beta x} + Re^{-\gamma x}\right) + n_0/\tau e^{-\beta x} + \alpha F_0 e^{-\alpha x} \\ &= \left(\left(\alpha^2 D - \alpha v_d - 1/\tau\right)P + \alpha F_0\right)e^{-\alpha x} + \left(\left(\beta^2 D - \beta v_d - 1/\tau\right)Q + n_0/\tau\right)e^{-\beta x} \\ &\quad (S13) \end{aligned}$$

This gives us

$$P = \frac{\alpha F_0}{1/\tau + \alpha v_d - \alpha^2 D}; \; Q = \frac{n_0/\tau}{1/\tau + \beta v_d - \beta^2 D} \qquad (S14)$$

For R we use the boundary condition at $x = 0$. We assume that electron concentration at $x = 0$ remains the same even at non-equilibrium

$$R = \frac{n_0(\beta v_d - \beta^2 D)}{1/\tau + \beta v_d - \beta^2 D} - \frac{\alpha F_0}{1/\tau + \alpha v_d - \alpha^2 D} \qquad (S15)$$

Justification of the Key Assumptions

To solve the continuity equation above, we made use of the following assumptions

Assumption #1: The width of the band bending region does not change with bias significantly and the electric field in the band bending region is given approximately by $E = (V_{bi} - V)/W$ where V is the applied bias.

Assumption #2: The electron density at the interface of QD and ZnO, $n(0)$, is nearly unchanged with current.

Justification for assumption #1:

We show that at equilibrium, there exists a discontinuity in electric field at $x = W$. For $0 < x < W$, E is constant and for $x > W$, $E = 0$. The discontinuity of E-field at $x = W$ means that there exists a sheet of negative charge at W. Physically, this negative charge resides in a single layer of ligand conjugated quantum dots. The solution at equilibrium also shows that nowhere else in the QD layer except at W contains any net charge. Note that there is a significant difference between the space charge in a p/n junction versus the sheet of charge in CQD layer. The space charge in the p/n junction is caused by donor or acceptor impurities, so the amount of charge is fixed (i.e. equal to the impurity concentration). For CQDs, no impurity is intentionally introduced as donors or acceptors and the amount of sheet charge can increase or decrease according to the bias voltage. In addition, the width of the sheet charge can also vary.

When a voltage bias is applied to the structure, there are two possible scenarios of how the charge is distributed within the QD layer: (i) the position of W does not change but the amount of sheet charge at W changes with the bias voltage, or (ii) both the amount of sheet charge at W and the width of the sheet charge change. We cannot determine either (i) or (ii) happens because the detailed characteristics depend on the density and distribution of the states for the ligand-QD structure. If the number of available states is large, the change in the applied bias will modulate

the amount of sheet charge without changing W (i.e. scenario (i)). If the number of available states for the ligand-QD structure is modest, then the charged region can be extended to the next ligand-QD layer. Since we are dealing with low (forward and reverse) bias and each QD layer is only 4nm thick, the position and width of the charged region under bias will change little from its equilibrium position under either scenario (i) or (ii). This explains why we use the approximate relation $E = (V_{bi} - V)/W$ where W is the position of the sheet charge at equilibrium.

A graphic representation for our assumption is shown in Fig. S1.

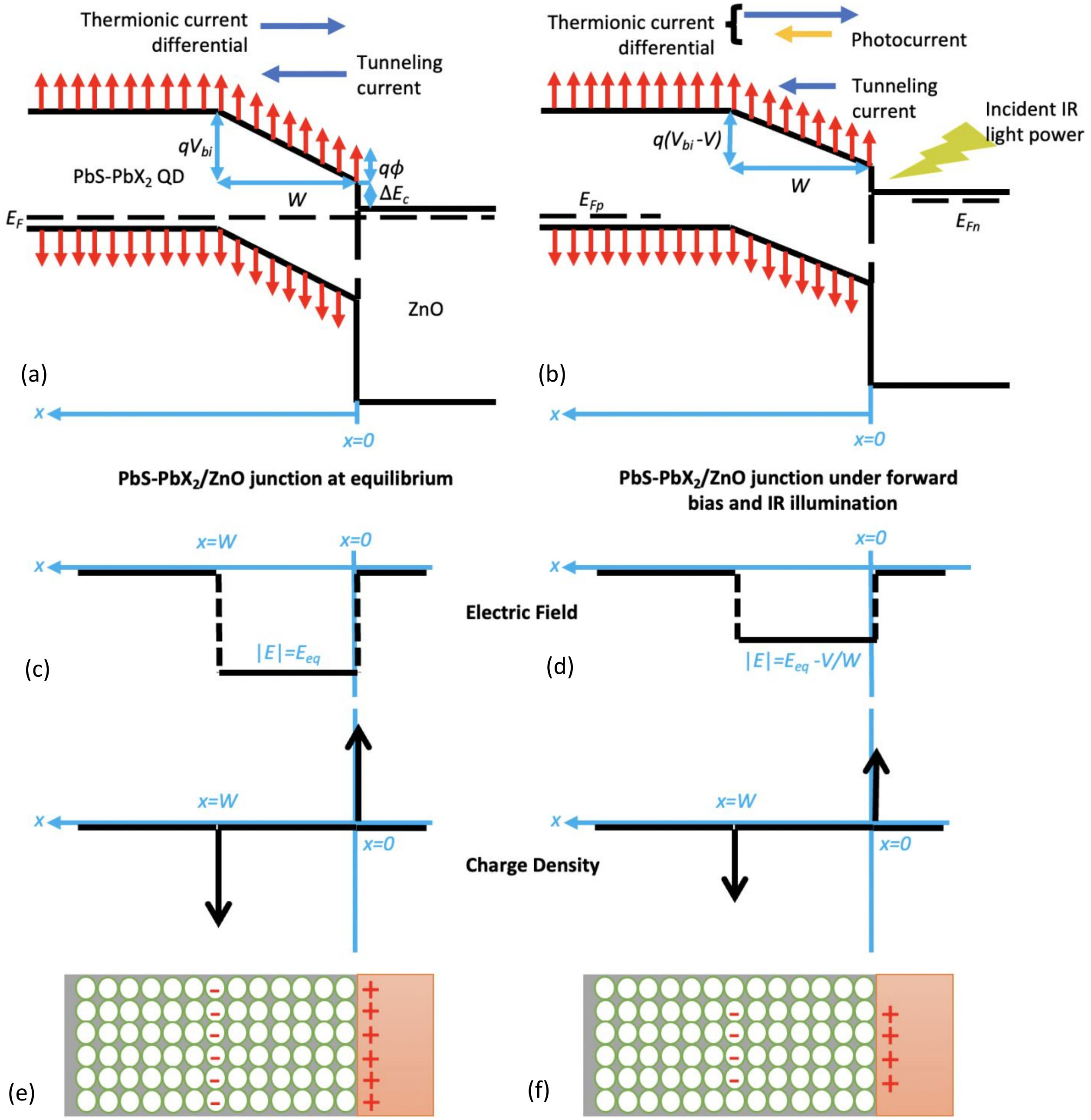

Figure S1. Physical Picture of the Model (a) Band bending at equilibrium. (b) Band bending under bias. (c) Electric field at equilibrium. (d) Electric field under bias. (e) Charge density at equilibrium. (f) Charge density under bias.

Justification for assumption #2: the value of electron concentration at the QD/ZnO interface is primarily determined by the doping level of ZnO (the electron transport layer) and the interface

states. The interface electron concentration is insensitive to the current density under low current condition.

Derivation of the Total Current Expression

Substituting (S12) in (S7),

$$J_n(x) = q\left[P(v_d - \alpha D)e^{-\alpha x} + Q(v_d - \beta D)e^{-\beta x} + R(v_d - \gamma D)e^{-\gamma x}\right] \qquad (S16)$$

Total current in the direction of PbS-QD to ZnO is be given by

$$J = -J_n(0) = q\left[D(\alpha P + \beta Q + \gamma R) - (P + Q + R)v_d\right] = q\left[D(\alpha P + \beta Q + \gamma R) - n_0 v_d\right] \qquad (S17)$$

It should be noted that the negative sign in front of $J_n(0)$ is due to our choice of x-coordinate direction, which is opposite to the conventional choice (p-side to n-side).

The term $\alpha P + \beta Q + \gamma R$ is evaluated as

$$\alpha P + \beta Q + \gamma R = \frac{\alpha^2 F_0}{1/\tau + \alpha v_d - \alpha^2 D} + \frac{n_0 \beta/\tau}{1/\tau + \beta v_d - \beta^2 D} + \frac{\gamma(\beta v_d - \beta^2 D)n_0}{1/\tau + \beta v_d - \beta^2 D} - \frac{\alpha\gamma F_0}{1/\tau + \alpha v_d - \alpha^2 D} = \frac{n_0\beta\left(1/\tau + \gamma(v_d - \beta D)\right)}{1/\tau + \beta v_d - \beta^2 D} + \frac{(\alpha^2 - \alpha\gamma)F_0}{1/\tau + \alpha v_d - \alpha^2 D} \qquad (S18)$$

Substituting (S18) in (S17)

$$J = q\left[\frac{n_0\beta D\left(1/\tau + \gamma(v_d - \beta D)\right)}{1/\tau + \beta v_d - \beta^2 D} - n_0 v_d\right] - \frac{q\alpha(\gamma - \alpha)DF_0}{1/\tau + \alpha v_d - \alpha^2 D} \qquad (S19)$$

The first term (in square brackets) is the dark current density.

$$J_{dark} = q\left[\frac{n_0\beta D\left(1/\tau + \gamma(v_d - \beta D)\right)}{1/\tau + \beta v_d - \beta^2 D} - n_0 v_d\right] = \frac{q n_0(\beta D - v_d)(1/\tau - \beta(\gamma D - v_d))}{1/\tau - \beta(\beta D - v_d)} \qquad (S20)$$

Shockley-like Simplification of the Model

We can simplify the term $(\gamma D - v_d)$ in (S20) by using the approximation $4D/\tau v_d^2 \ll 1$.

$$\gamma D - v_d = \sqrt{\left(\frac{v_d}{2}\right)^2 + \frac{D}{\tau}} - \frac{v_d}{2} = \frac{v_d}{2}\left(\sqrt{1 + \frac{4D}{\tau v_d^2}} - 1\right) \approx \frac{D}{\tau v_d} \qquad (S21)$$

Substituting (S21) in (S20),

$$J_{dark} = \frac{q n_0}{\tau v_d}\left(\frac{(\beta D - v_d)^2}{\beta(\beta D - v_d) - 1/\tau}\right) \qquad (S22)$$

Focusing on the term $(\beta D - v_d)$

$$\beta D - v_d = v_R\left(e^{-B/E_{eq}} - e^{-B/E}\right) = v_R e^{-B/E}\left(\exp\left(\frac{B(E_{eq} - E)}{E \times E_{eq}}\right) - 1\right) = v_d\left(\exp\left(\frac{BV}{EV_{bi}}\right) - 1\right) \qquad (S23)$$

where V is the applied bias and V_{bi} is the built-in voltage in CQD layer. The electric field (assumed constant) is given by $E = (V_{bi} - V)/W$ where W is the width of the band-bending region (given by $W = V_{bi}/E_{eq}$). As V varies from 0 to V_{bi}, E varies from E_{eq} to 0. Therefore, we can approximate (S23) by using the average value of electric field ($\langle E \rangle = E_{eq}/2$).

$$\beta D - v_d \approx v_d\left(\exp\left(\frac{2BV}{E_{eq} V_{bi}}\right) - 1\right) \qquad (S24)$$

Finally, substituting (S24) in (S22) and assuming $\beta(\beta D - v_d) \gg 1/\tau$,

$$J_{dark} = \frac{qn_0}{\tau\beta}\left(\exp\left(\frac{2BV}{E_{eq}V_{bi}}\right) - 1\right) = \frac{n_0 kT}{\tau E_{eq}}\left(\exp\left(\frac{2BV}{E_{eq}V_{bi}}\right) - 1\right) \qquad (S25)$$

For photocurrent, we can assume v_d to be close to the equilibrium value at low bias.

$$J_{photo} = -\frac{q\alpha(\gamma_0 - \alpha)DF_0}{1/\tau + \alpha v_{d0} - \alpha^2 D} \qquad (S26)$$

where v_{d0} is the v_d at equilibrium and γ_0 is the corresponding γ. Comparing (S25) and (S26) to the Shockley-diode equation ($J = J_0\left(e^{qV/\eta kT} - 1\right) - J_L$), we have the following expressions for the reverse saturation current density (J_0), effective ideality factor (η) and internal quantum efficiency (QE).

$$J_0 = \frac{n_0 kT}{\tau E_{eq}}; \eta = \frac{qE_{eq}V_{bi}}{2BkT}; QE = \frac{\alpha(\gamma_0 - \alpha)D}{1/\tau + \alpha v_{d0} - \alpha^2 D} \qquad (S27)$$

List of Parameters

For figures 1-4, the following parameter values are used unless a parameter is varied to study its dependence.

Table S1. List of parameters used in the model.

Electron effective mass (m_n^*)	$0.17m_0$
Quantum dot diameter + Ligand length (d)	4 nm
Tunneling barrier height (ϕ)	0.1258 V
Minority carrier lifetime (τ)	5 µs

Built-in voltage (V_{bi})	1 V
Electron density at the heterojunction (n_0)	10^{15} cm $^{-3}$
Absorption coefficient (α)	8.5×10^5 cm $^{-1}$
Area-normalized series resistance (R_s)	177 Ωcm^2
Light reflection loss	0
<u>Model Calculated Parameters</u>	
Equilibrium electric field (E_{eq})	4.2×10^5 V/cm
Zero-bias drift velocity (v_{d0})	3.3×10^5 cm/s
Effective diffusion coefficient (D)	0.0203 cm^2/s
Width of band bending region (W)	23.81 nm

CQD Synthesis and Device Fabrication

Materials: lead(II) oxide (PbO) (99.999%), oleic acid (OA) (90%), bis(trimethylsilyl)sulfide (TMS_2) (98%), zinc acetate dihydrate(>98%), potassium hydroxide (KOH), Ammonium acetate (NH_4Ac), lead(II) iodide (PbI_2), oleic acid (OA) (90%), lead(II) bromide ($PbBr_2$), 1,2-ethanedithiol (EDT) (98%) and 1-octadecene (ODE) (90%) were purchased from the Sigma-Aldrich Co. All reagents were used without further purification.

Synthesis of ZnO NPs and PbS QDs: ZnO NPs were synthesized using a slightly modified method from a previous study[1]. Zinc acetate dihydrate (2.95 g) in methanol (125 mL) was added to a 250 mL three-neck flask. The solution was heated to 60 °C and KOH solution (1.48 g in 65 mL of methanol) was slowly injected into the flask. The mixture was maintained at 60 °C for 2.5 h and then cooled to 25 °C. The obtained ZnO NPs were purified thrice using pure methanol and collected in chloroform (70 mg mL^{-1}). PbS QDs were synthesized using the hot-injection method described in a previous report[2]. OA (2.8 mL), PbO (0.9 g), and ODE (20 mL) were added to 100 mL three-neck flask and degassed for 2 h at 110 °C. Next, the mixture was heated to 120 °C under a nitrogen atmosphere and the sulfur precursor (240 μL of TMS_2 in 8 mL of ODE) in a 10 mL syringe was injected into the flask. The synthesized QDs were purified three times using acetone and ethanol. Finally, the QDs were dispersed in octane (50 mg mL^{-1}).

Ligand exchange process:

In order to fabricate PbS–PbX_2 layer, a solution exchange process was conducted by following a previously reported method with slight modifications[3]. Lead halides (0.46 g of PbI_2 and 0.07 g of $PbBr_2$) and NH_4Ac (0.03 g) were dissolved in DMF (10 mL) and vigorously mixed with the synthesized QDs (10 mL, 10 mg mL^{-1}) solution for 2 minutes. PbS QDs were completely transferred to the DMF phase and washed three times with pure octane. After the exchange process, CQDs were precipitated by centrifugation. After the vacuum-drying process, QDs were collected using butylamine (250 mg mL^{-1}). A solid ligand exchange was performed for PbS–EDT layer by a previously reported method[2]. As-synthesized PbS QD (50 mg mL^{-1}) was spin-coated and soaked with 0.02 vol% EDT acetonitrile-based solution for 30 s. After ligand exchanges, the film was washed with pure acetonitrile three times to remove residues.

Fabrication of the NIR photodetector:

An indium tin oxide (ITO) glass was cleaned with acetone, isopropanol, and deionized water for 10 minutes *via* sonication. The ITO glass was treated with UV-ozone for 15 min and then ZnO layers were spin-coated on the substrate with 2500 rpm for 30 s. The solution-exchanged PbS-PbX_2 QDs were then spin-coated onto the ZnO thin films at 2500 rpm for 30 s. Next, the desired thickness of PbS–EDT layers was achieved by performing the solid ligand exchange processes twice. Finally, a 100-nm-thick Au top electrode was deposited on the electron transport layers *via* thermal evaporation. The area of the device was 4 mm^2.

Additional Experimental Data

Here we provide more experimental data produced by different structures and fabrication methods to exhibit the validity of our model. The added data include: smaller devices (250um diameter) with the ITO/PbS-EDT/ZnO structure and devices having a sputtered ZnO layer instead of ZnO nanoparticles. We have also used PbS QDs synthesized in our lab and purchased from commercial vendors. These additional experimental data shown in Fig. S2 help demonstrate the general applicability of the model to different CQD device structures and materials.

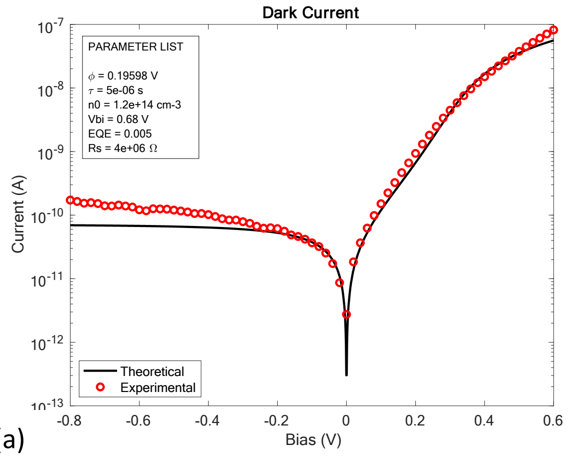

(a)

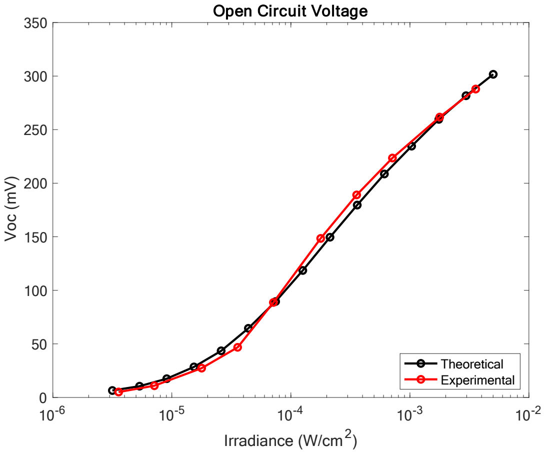

(b)

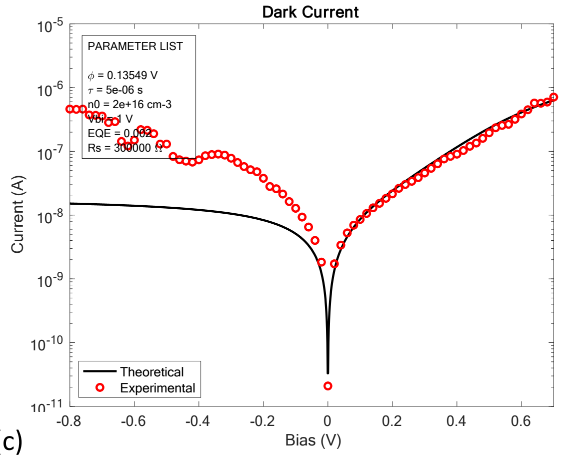

(c)

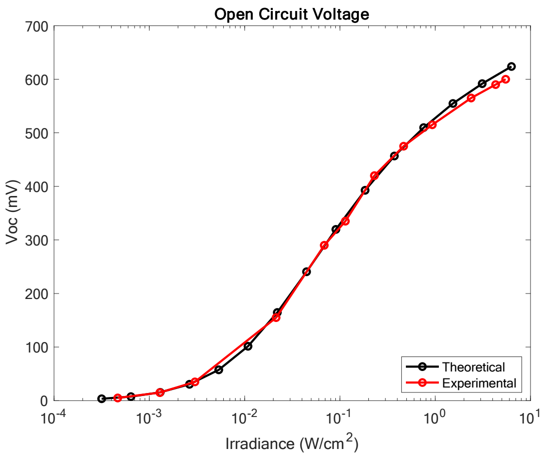

(d)

Figure S2. Additional Data for Comparison Between the Model and Experiment (a) dark current for PbS-EDT(lab-synthesized)/ZnO(sputtered) heterojunction. (b) open-circuit voltage for PbS-EDT(lab-synthesized)/ZnO(sputtered). (c) dark current for PbS-EDT(commercial)/ZnO(sputtered) heterojunction. (d) open-circuit voltage for PbS-EDT(commercial)/ZnO(sputtered).

REFERENCES

(1) Woo, H. K.; Kang, M. S.; Park, T.; Bang, J.; Jeon, S.; Lee, W. S.; Ahn, J.; Cho, G.; Ko, D.-K.; Kim, Y.; Ha, D.-H.; Oh, S. J. Colloidal-Annealing of ZnO Nanoparticles to Passivate Traps and Improve Charge Extraction in Colloidal Quantum Dot Solar Cells. *Nanoscale* **2019**, *11* (37), 17498–17505. https://doi.org/10.1039/C9NR06346C.

(2) Jung, B. K.; Woo, H. K.; Shin, C.; Park, T.; Li, N.; Lee, K. J.; Kim, W.; Bae, J. H.; Ahn, J.; Ng, T. N.; Oh, S. J. Suppressing the Dark Current in Quantum Dot Infrared Photodetectors by Controlling Carrier Statistics. *Adv. Opt. Mater.* **2022**, *10* (2), 2101611. https://doi.org/10.1002/adom.202101611.

(3) Sukharevska, N.; Bederak, D.; Goossens, V. M.; Momand, J.; Duim, H.; Dirin, D. N.; Kovalenko, M. V.; Kooi, B. J.; Loi, M. A. Scalable PbS Quantum Dot Solar Cell Production

by Blade Coating from Stable Inks. *ACS Appl. Mater. Interfaces* **2021**, *13* (4), 5195–5207. https://doi.org/10.1021/acsami.0c18204.
